# Preclinical evaluation of anti-*Helicobacter spp.* activity of *Hippocratea celastroides* Kunth and its acute and sub-acute toxicity

**DOI:** 10.1186/s12906-016-1412-6

**Published:** 2016-11-08

**Authors:** Griselda García-Alonso, Antonio Monroy-Noyola, Armando Contreras-Arellano, José Fernando Mariscal-Durand, Yolanda Gálvez-Molina, Alejandro Vázquez-Velázquez, Sara García-Jimenez, Pablo Nuñez, Alexandre Cardoso-Taketa, María Luisa Villarreal

**Affiliations:** 1 Centro de Investigación en Biotecnología, Av. Universidad 1001. Col. Chamilpa, Cuernavaca, 62209 Morelos México; 2 Facultad de Farmacia, Universidad Autónoma del Estado de Morelos, Av. Universidad 1001. Col Chamilpa, Cuernavaca, 62209 Morelos México; 3 Consultorio Médico de Diagnóstico Gastroenterológico y Quirúrgico, Domingo Diez, Miraval, 62270 Cuernavaca, Morelos México; 4 Hospital Veterinario Animal’s, Av. Emiliano Zapata 833, Cuernavaca, Morelos México; 5 Hospital General de Cuernavaca “Dr. José G Parres”, Domingo Diez, Miraval, 62270 Cuernavaca, Morelos México

**Keywords:** Antimicrobial, Anti-*Helicobacter spp*, *Hippocratea celastroides*, Mexican traditional medicine

## Abstract

**Background:**

*Hippocratea celastroides* Kunth, commonly known as “cancerina”, is used in Mexican Traditional Medicine for the treatment of gastric and intestinal infections, systemic and skin inflammation, injuries and gastritis. The aim of this research was to assess the anti-*Helicobacter pylori* activities of hydro-ethanolic root-bark extracts from *Hippocratea celastroides* Kunth in naturally infected dogs, after testing their acute and subacute toxicities in mice.

**Methods:**

To determine in vivo acute toxicity, a hydro-ethanolic extract was obtained and administered orally in female and male Balb-C mice, at doses ranging from 2000 to 5000 mg/kg. For the subacute study, a hydro-ethanolic extract was given to male and female Balb-C mice at doses ranging from 200 to 2000 mg/kg body weight. The animals were observed daily over a period of 42 days for signs of toxicity. In the pre-clinical anti-*Helicobacter spp.* assay, 60 dogs were included. Eighteen and 19 dogs for the experimental and control groups respectively, concluded the study. The experimental treatment consisted of *H. celastroides* hydro-ethanolic extract and the control treatment of amoxicillin-clarithromycin-omeprazole.

**Results:**

Oral LD_50_ (lethal dose 50) values for hydro-ethanolic extract were indeterminable at the highest tested doses. Under the subacute administration, neither mortality nor any sign of toxicity were observed when the hydro-ethanolic extract was administered. There were no significant alterations in biochemical parameters. The prevalence of *Helicobacter* spp*.* infection in dogs was 97.1 % for the experimental group and 100 % for the control group. Effectiveness was of 33.3 and 55 % in the experimental and control group respectively. The oral administration of *H. celastroides* was well-tolerated and safe.

**Conclusion:**

The root-bark of *H. celastroides* produced no signs of toxicity, and manifested pharmacological activity that indicated the possibility of an alternative treatment for *H. pylori* infection. Effectiveness is still low so it is necessary to continue research.

**Electronic supplementary material:**

The online version of this article (doi:10.1186/s12906-016-1412-6) contains supplementary material, which is available to authorized users.

## Background

*Hippocratea celastroides* Kunth*,* a shrub-like vine that is widely distributed throughout Mexico, grows in tropical deciduous forests [1]. Its popular names in Mexico are “cancerina”, “barajilla”, “barajita”, “bejuco de piojo”, “cucaracho”, “hierba del piojo”, “ixcate”, “ixcatecimarron”, “izcate blanco”, “mata piojo”, “piojoso”, “quina” [2, 3]. In the state of Mexico, *H. celastroides* is used as a purgative, a stomach antiseptic, a general de-wormer and also an acaricide [4–6]. In the state of Morelos, the infusion is used for the treatment of gynecological conditions, and topically for cuts and bruises, whereas the baked root-bark is used to treat topical and internal inflammation, as well as infections, injuries and gastritis [3, 7].

According to phytochemical investigations reported for this species, alditol galactitol was identified from the roots [8]. Celastroidine A (C_50_H_74_O_5_) was identified as a Diels–Alder adduct of a triterpene plus a diterpene, whereas Celastroidine B (C_40_H_60_O_4_) was identified as a dimer of a beyerane diterpene [9]. Toxicity and anti-feeding properties of Celastroidine A and B were examined as a control against the stored grain pest *S. zeamays*. Celastroidine A inhibited the feeding of the insect 88.7 % with a mortality increase of 2 %, whereas B showed 9.6 % anti-feeding inhibition with a mortality of 5.2 % [2].

In our previous study, when acute ulcers were induced in mice through oral administration with absolute EtOH, *H. celastroides* MeOH root extract evidently provided gastro-protective activity [10]. In this same investigation, the topical anti-inflammatory action of the extract using the ear acute edema mice model was recorded. The root extract showed cytotoxic activity against nasopharyngeal (KB) and breast (MCF-7) cancer cell lines, but non-toxic selectivity towards a normal fibroblast cell line (HFS-30). The MeOH extract exhibited in vitro anti-*H. pylori* activity and registered a MIC value of 7.8 μg/mL [10].

The discovery of *Helicobacter pylori* in human beings and its relationship to gastritis, peptic ulcer disease, gastric adenocarcinoma, and mucosa-associated lymphoid tissue (MALT) lymphoma [11, 12], has encouraged investigation of the incidence, clinical significance and treatment of *Helicobacter spp.* infection in domestic pets, specifically dogs. The presence of *Helicobacter spp.* in gastric canine mucosa provokes mixed infections caused by various *Helicobacter* species (*Helicobacter pylori*, *Helicobacter felis*, *Helicobacter bizzozeronii*, *Helicobacter candidatus*, *Helicobacter heilmannii*, *Helicobacter cynogastricum,* and *Helicobacter salomonis* [13–16]. In dogs, the above spiral-shaped bacteria are found in the gastrointestinal tract. According to numerous studies they are present in between 62.7 and a 100 % of healthy dogs and dogs with signs of gastritis, including client-owned dogs and other dogs, euthanized for various reasons [17–21]. *H. felis* has been associated with active chronic gastritis and *H. bizzozeronnii* with duodenal and gastric ulcers [22]. Naturally occurring *Helicobacter,* can also colonize the intestinal crypts leading to lymphocytic enteritis and canine inflammatory bowel disease, often associated with diarrhea, gastro esophageal reflux and vomiting [23, 24]. There is documented evidence that domestic animals are a source of infection for human beings [22]. The species that colonize the human gastric mucosa are *H. felis, H. salomonis, H. bizzozeronii* and *Candidatus Helicobacter heilmannii* [22, 25]. *Helicobacter spp.* transmission mechanisms are fecal-oral and oral-oral. Different studies suggest that direct contact with pets, and poor hygiene conditions, including contaminated food and water, may be determining factors for transmission between humans and animals [26–29]. Treatment prescribed to eradicate *Helicobacter spp.* in dogs is the same current therapy schema prescribed to eradicate *Helicobacter pylori* in humans. Preferred treatment in Mexico, as well as in other countries is represented by a triple therapy, which includes an acid secretion inhibitor (omeprazole), in combination with two antibiotics (amoxicillin and clarithromycin) [30–34]. However, there is also a problem of antimicrobial resistance, as well as easy re-infection or recurrence for various reasons, situations that have been published in several studies [26, 35, 36].

The frequent occurrence of gastric *Helicobacter* in pets, the risk of it being transmitted to human beings and its bacterial resistance have motivated this investigation to determine the effectiveness, safety and tolerability of hydro-ethanolic *Hippocratea celastroides* root extract to combat this bacterial infection. Firstly, we evaluated the acute and subacute toxicological effects of this plant extract on mice, and then we determined the prevalence of *Helicobacter spp.* in a population of naturally infected dogs.

## Methods

### Plant material

*Hippocratea celastroides* Kunth (Hippocrateaceae) root-barks were collected in June 2011 in Yautepec, Morelos, Mexico. The plant was identified by Rolando Ramirez from the Herbarium of CIB (Centro de Investigaciones Biológicas) at the Universidad Autónoma del Estado de Morelos (UAEM), where a botanical voucher No. 26447 was deposited for reference. Plant material was dried under dark conditions for a period of two months. A 70 % hydro-ethanolic extract of *Hippocratea celastroides* using the root barks was prepared by MIXIM Laboratories (http://www.labmixim.com/espanol/historia.html), code number 1901097. An HPTLC analysis of *H. celastroides* extract to detect the presence of alkaloids and triterpenes was performed (Additional file 1).

### In vivo toxicological evaluation

The rodents were obtained from the animal laboratory of INSP (Instituto Nacional de Salud Pública), Cuernavaca, Morelos, Mexico. Male and female Balb-C mice (18 ± 25 g) of 6–8 weeks were used. All animals were clinically healthy and maintained under regular husbandry conditions; 23 ± 2°, 12 h light dark cycle with *ad libitum* access to water and standard rodent chow. In order to become familiarized with environmental and handling conditions, all animals were introduced to translucent animal cages and handled daily for 1 week, prior to experimentation.

#### Acute toxicity

Rodents were separated into five groups; ten rodents in each group, ten females and ten males. These comprised the control group and four experimental groups, each of which received different doses of the hydro-ethanolic extract from *H. celastroides* (2000, 3000, 4000 and 5000 mg/kg of body weight with an oral single dose diluted in 0.9 % saline solution). The control group received 0.9 % saline solution at an equivalent volume. The rodents were deprived of food and water 2 h prior to administration of the extracts. The solutions were administered using a metallic cannula. Observations were made and systematically recorded after 1, 2 and 4 h of extract administration. Visual observations included skin changes, modifications in respiratory patterns, motility, diarrhea, behavioral pattern, convulsions and death. Animal mortality and survival were recorded daily for 14 days subsequent to extract administration, after which they were sacrificed by decapitation.

#### Sub-acute toxicity

For the assay, four groups (ten rodents each, five female and five male) to test doses of 200, 1000, 2000 mg/kg and the control group were formed in a similar way. Six weeks old rodents were deprived of food but not of water 2 h prior the administration of the tested substances. A daily dose of hydro-ethanolic extract from *H. celastroides* was administered for 28 days to each group. The hydro-ethanolic extract solution was prepared every day by dissolving the crude extract in 0.9 % saline solution and then administered using a metallic cannula. The control group was given only the vehicle using the same route and volume. All rodents were observed daily, as well as 14 days after having finished the extract administration to detect signs of toxicity or behavioral alterations during the experimental period. On day 42, all rodents were sacrificed by decapitation, and the organs (brain, liver, kidney, heart, pancreas, stomach, lungs, and intestine) were collected for macroscopic observation. The serum aspartate transaminase (AST), as well as urea and creatinine were determined.

### Preclinical anti-*Helicobacter spp.* assay

#### Subjects

In this study, we included 60 adult dogs, 6–32 kg. The controlled preclinical trial was conducted at the APAC (Asociación Protectora de Animales Philip Kahan) shelter for dogs, and at the “Animal’s” Veterinary Hospital. Animals included symptomatic and asymptomatic dogs of both sexes. Excluded animals were those with cardiac hepatic or renal illness, pregnant, with additional infections, or taking ulcerogenic antibiotic or anti-secretory treatments. The treatments were assigned according to the section assigned in the dog shelter; region A was assigned for experimental treatment and section B for the control group. Thirty five dogs were enrolled in the experimental group and 25 dogs in the control group, 16 and 6 dogs in the experimental and control groups respectively were excluded because they had been given up for adoption, or the owners decided to suspend the anti-*Helicobacter* treatment. None of the dogs involved suffered adverse effects. At the end, the experimental group included only 18 dogs (one dog was negative to *Helicobacter* infection) and the control group included only 19 dogs.

#### Study intervention

The experimental group received 500 mg of the hydro-ethanolic extract from *Hippocratea celastroides* root in capsules of 500 mg every 12 h for a period of 12 weeks, and the control group received the triple schema of 500 mg amoxicillin, 500 mg clarithromycin and 20 mg omeprazole every 12 h for a period of 7 days.

#### Study protocol

To perform the upper digestive endoscopy, the anesthetic medication consisting of a mixture of 1 mg/kg Xilacine and 7 mg/kg Zoletil 100 (Tiletamine/zolazepam), was injected intravenously to all dogs involved. The same gastroenterologist, using an Olympus GIF-130 gastroscope, performed all the endoscopies. The first endoscopy for each dog was performed prior to initial treatment in order to make the *Helicobacter spp.* infection diagnosis, and a second endoscopy was performed at the end of the assigned treatment to verify eradication. The samples collected in both endoscopies were from fundus, antrum and pre-pyloric regions. One sample from each stomach region was immediately immersed in a 10 % formol solution in order to implement histopathologic exams by staining with haematoxylin-eosin and giemsa. The dogs in the experimental group were observed weekly throughout the research period and daily for the control group, for the purpose of checking possible adverse effects. One day following conclusion of therapy, the animals were submitted to a new upper digestive endoscopy to take gastric mucosal biopsies from fundus, antrum and pre-pyloric regions, in order to verify bacterial eradication. To investigate the safety of *H. celastroides* extract and the triple schema, blood samples were obtained prior to initiation of treatment, and again when the second endoscopy was performed.

#### Outcome measures

The bacillary presence or absence of *Helicobacter spp* was confirmed at the end of the assigned treatment with the stain haematoxylin-eosin and giemsa. Absence of bacillus in samples from the three regions studied was considered as signifying eradication. Side effects were registered weekly to assay tolerability by means of a table assigned to each dog for internal use at the veterinary hospital. Safety was determined by following the plasma biochemical levels of urea, creatinine, ALT and AST, prior to treatment initiation and at the end of the assigned treatment.

### Statistical analysis

In the sub-acute toxicity study, body weight data were expressed as the mean ± standard error of the mean (S.E.M.). All values with normal distribution and homogeneity among variances were analyzed by one way ANOVA followed by Dunnett’s multiple comparison tests. Analysis of effectiveness was performed using chi-square test. Serum values of biochemical parameters were analyzed by using *t*-test; the results are expressed as mean ± standard deviation of the mean. The Graph Pad Prism Statistics Software program was used. A probability level of less than 0.05 was considered significant.

## Results

In the toxicity assays, the oral LD_50_ values for hydro-ethanolic extract were not determined as none of the rodents exhibited any toxicological symptoms such as diarrhea, skin changes, alterations in respiration, motility, and behavioral patterns, convulsions or death for up to the highest assayed dose (5000 mg/kg). LD_50_ values higher than 5000 mg/kg are considered non-toxic according to the GHS (Globally Harmonized System).

**Fig. 1:**
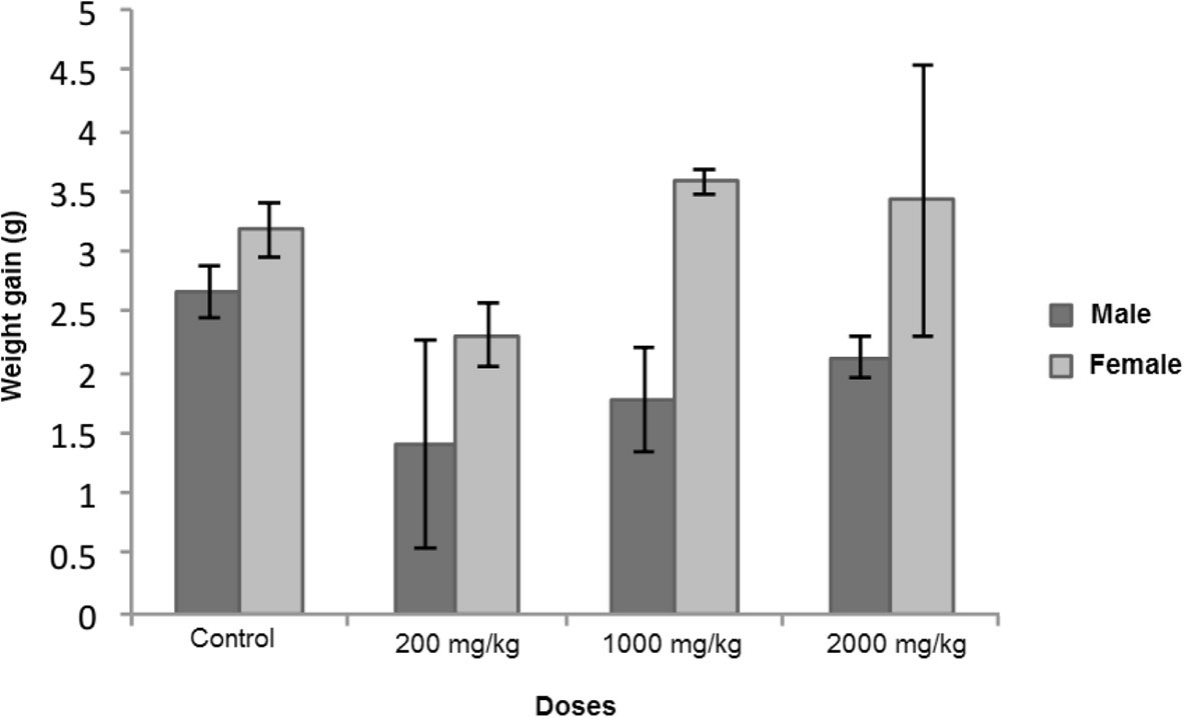
Effect of hydro-ethanolic root-bark extract of *Hippocratea celastroides* on weight gain at 200, 1000, and 2000 mg/kg body weight administered daily for 28 days. Foot note.- (Mean ± SE) *n* = 10, *p* < 0.05 Dunnett’s. Non-significant changes were observed compared to control

In the sub-acute toxicity treatment using the hydro-ethanolic extract, the differences in weight gain at day 28 between control and experimental rodents, both male and female, showed no statistical differences (Fig. 1). No morbidity or mortality was observed during the first 28 days, or in the following 14. Blood chemical analyses were performed in order to evaluate any toxic effects on the kidney (urea and creatinine) and liver (AST) function. Table 1 shows the results of serum parameters for animals to which *H. celastroides* was administered. Regarding AST levels, the extract administered at a dose of 2000 mg/kg body weight over a 28 days period showed no significant difference when compared to the control group. Although there was a significant difference at a dose of 200 mg/kg, the value fell within the normal range and had no clinical relevance. A significant difference was detected between the doses of 200 and 1000 mg/kg regarding urea level; although, these values also fell within the normal ranges [37]. Creatinine values also fell within the normal range, indicating that at the end of the treatment both liver and kidney were healthy.

**Table 1:** Blood chemistry values with the hydro-ethanolic extract of *H. celastroides* in the subacute toxicity assay

	Control	200 mg/kg	1000 mg/kg	2000 mg/kg
AST (U/L)	41.07 ± 4.1*	13.62 ± 3.6	31.5 ± 3.6*	38.28 ± 5.9*
Urea	23.1 ± 0.80*	27.77 ± 1.13	26.9 ± 1.38	23.75 ± 0.77*
Creatinine	0.2 ± 0.03*	0.26 ± 0.03*	0.21 ± 0.01*	0.25 ± 0.03*

*n* = 10 (for control and experimental groups). *AST* aspartate aminotransferase. *(*P* < 0.05)

**Fig. 2:**
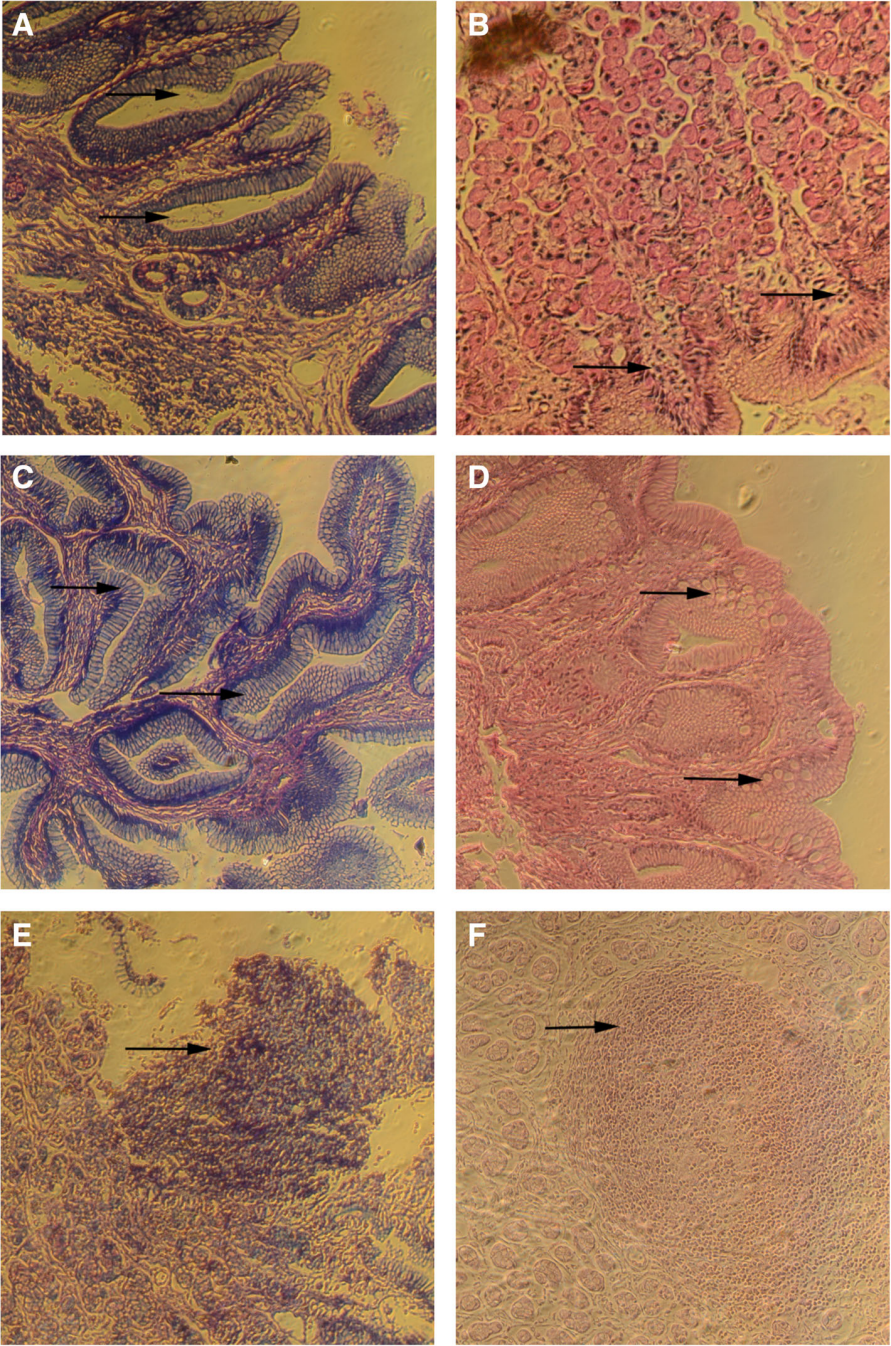
Lesions of gastric mucosa of dogs in the pre-clinical evaluation of anti-*Helicobacter spp.* activity of *H. celastroides*. Foot note.- Presence of *Helicobacter spp.* intraglandular (**a**); mononuclear inflammatory infiltrates and edema in the chronic superficial gastritis (**b**); foveolar hyperplasia in the chronic chemical gastritis (**c**); intestinal metaplasia in the chronic atrophic gastritis (**d**); presence de lymphoid follicle in the chronic follicular gastritis (**e** and **f**), indicated by *arrows*

**Fig. 3:**
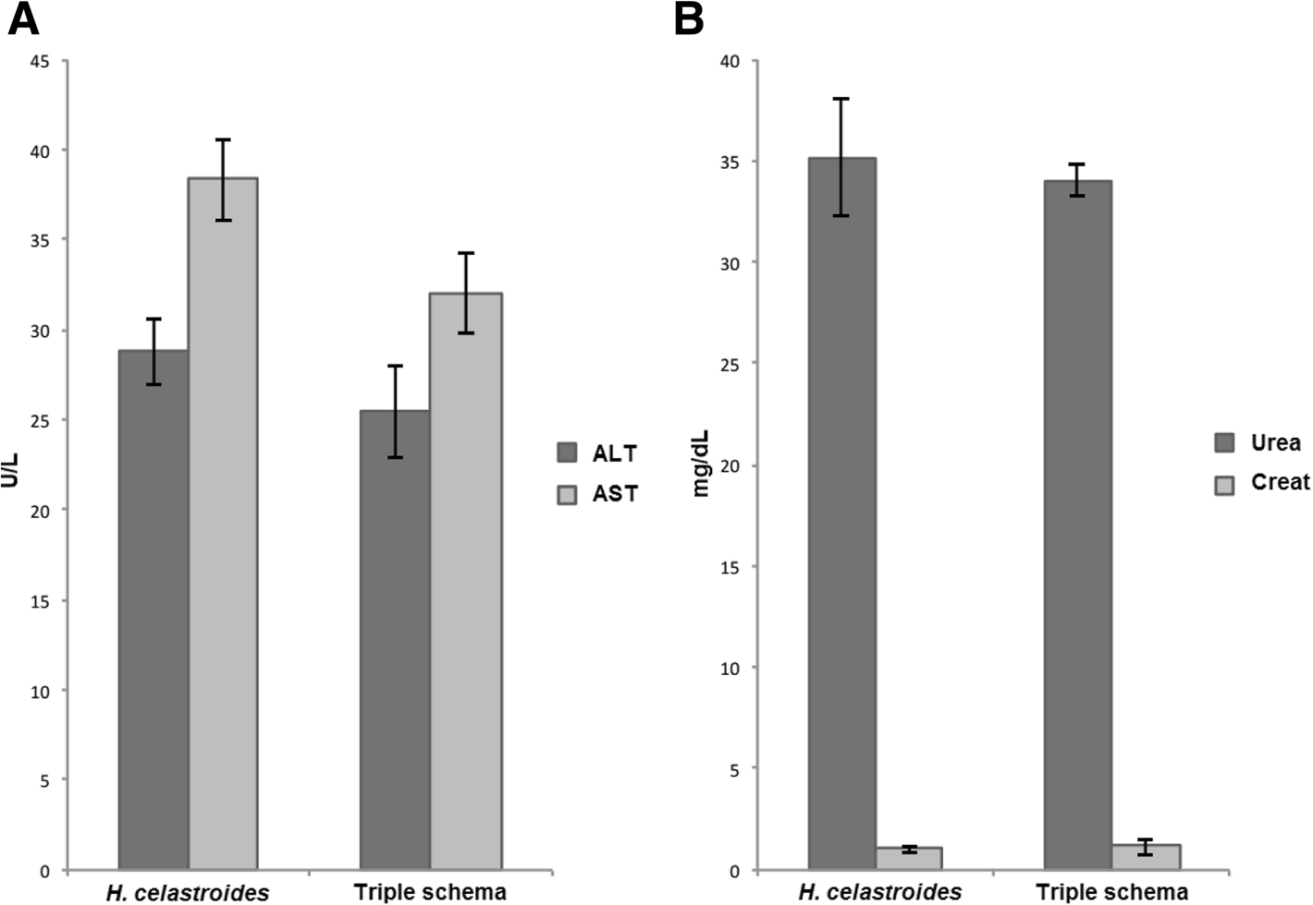
Blood chemistry values with the hydro-ethanolic extract of *H. celastroides* and triple schema in the therapeutic safety analysis. Foot note.- **a** ALT = alanine aminotransfesare, AST = aspartate aminotransferase. **b** Urea, and Creat = creatinine. Values are expressed as mean ± SE, *n* = 10. *p* < 0.05 *T* test. Non-significant difference was observed between both groups

The prevalence of *Helicobacter spp.* infection in the canine population studied was 97.1 and 100 % for the experimental and control groups respectively, referring to the initial number of dogs included in the study (35 and 25 in the experimental and control groups respectively). Only one dog was healthy. The classification of lesions was made according to the Updated Sydney Classification System. Mean lesions reported, prior to the treatment assigned for both groups, included chronic superficial gastritis, chronic follicular gastritis, chronic chemical gastritis, corporal chronic atrophic gastritis, and multifocal chronic atrophic gastritis (Table 2). Figure 2 shows the presence of *Helicobacter spp.* intraglandular (A); it is also possible to observe the mononuclear inflammatory infiltrates and edema in the chronic superficial gastritis (B); foveolar hyperplasia in the chronic chemical gastritis (C); intestinal metaplasia in the chronic atrophic gastritis (D); and the presence of lymphoid follicle in the chronic follicular gastritis (E and F). Mean clinical signs found in symptomatic dogs included diarrhea, vomiting, loss of weight and halitosis; all of which were eliminated during the first week of both treatments. The analysis for effectiveness showed 33.3 and 55 % eradication for the experimental (*H. celastroides* extract) and control group (amoxicillin-clarithromycin-omeprazole) respectively, without any significant difference between the two groups (Table 3). Regarding the overall tolerability of interventions, only 6 dogs in the control group experienced mild effects (diarrhea), so it was not necessary to exclude them from the study. The therapeutic safety (determined through urea, creatinine, AST, and ALT values) was 84.2 and 80 % in the experimental and control groups respectively, without significant differences (Table 4). Figure 3 shows the values for urea, creatinine, AST and ALT with non-significant difference in each media between both groups. The normal ranges for biochemical parameters in the literature reported for dogs are: AST 12–60 U/L [38, 39], ALT 10–100 U/L [39, 40], urea 21–60 mg/dL [41] and creatinine 0.5–1.6 mg/dl [41]. According to these data, our results show values out of the normal range in three dogs from the experimental group and four dogs from the control group; however, no clinical significance was evident, so the dogs did not require additional treatment and were observed for future alterations.

**Table 2:** Proportion of gastric mucosal lesions in dogs at baseline (experimental and control groups)

Histopathological diagnosis	f	rf
Chronic superficial gastritis	25	41.6
Chronic follicular gastritis	18	30
Chronic chemical gastritis	12	20
Chronic atrophic corporal gastritis	2	3.3
Chronic atrophic multifocal gastritis	2	3.3
Normal gastric mucosa	1	1.6

*n* = 60, *f* absolute frequency, *rf* relative frequency (%)

**Table 3:** *Helicobacter spp.* eradication using *H. celastroides* extract and triple schema (amoxicillin-clarithromycin-omeprazole)

	f (rf)	Chi^2^ (*P* value)
*H. celastroides* (*n* = 18)	6 (33.3)	
Triple schema (*n* = 20)	11 (55)	0.0899

*f* absolute frequency, *rf* relative frequency (%)

(*P* < 0.05)

**Table 4:** Safety therapeutic of *H. celastroides* and triple schema treatments

	f (rf)	Chi^2^ (*P* value)
*H. celastroides* (*n* = 19)	16 (84.2)	
Triple schema (*n* = 20)	16 (80)	0.3660

*f* absolute frequency, *rf* relative frequency (%)

Absence of any urea, creatinine, aspartate transaminase and alanine transaminase abnormal values

## Discussion

Considering toxicity parameters, no rodent death was recorded in either control or treatment groups during acute toxicity. We can thus conclude that *H. celastroides* root-bark is non-toxic with regard to the threshold for toxic substances (6 g/kg) stipulated by the GHS (Global Harmonized System). *H. celastroides* can be categorized as a non-observed-adverse-level (NOAEL) crude drug that acts safely under current normal usage [42, 43]. With respect to the assay of subacute toxicity, the hydro-ethanolic extract of *H. celastroides* root-bark presented no evidence of toxicity. The level of AST with the maximal dose of the plant extract (2000 mg/kg) had a mean of 38.28 ± 5.91 U/l, a low value when compared to other toxicological studies that indicate hepatic damage, for example the administration of CCl_4_, a potent chemical hepatotoxic that causes hepatocellular damage with markedly elevated activities of serum enzymes (mean 968.58 ± 439.52 U/L; >2000 U/L) [44, 45]. Some studies in the literature have described how pre-induction with 50 % (*v/v*) ethanol provoked a significant elevation of serum AST levels (902.8 ± 16.7) [45]. In relation to the kidney function tests for creatinine and urea [46], the results indicate that the rodents’ kidneys were not affected with the highest administered dose (2000 mg/kg). There were significant differences between the doses of 200 and 1000 mg/kg in terms of urea level; nevertheless, these values also fell within normal ranges [37]. The prevalence of *Helicobacter spp.* infection in dogs that we reported (98.1–100 %) is similar to that reported for other countries; 95–100 % Finland [18], 87–100 % USA [47], 61–99 % Germany [17], 85.1 % Poland [21] and 78.4–82.3 % in Korea [20]. Taking care of the zoonotic potential, this fact indicates the importance of the health problem for both humans and pets. In relation to the mean lesions found in the gastric mucosa of the dogs examined, our results concur with other studies that report the frequent occurrence of gastritis in dogs, mainly superficial gastritis, chronic active gastritis and lymphoid follicles [17, 19, 48, 49]. When compared to human studies, the lesions found in *H. pylori* infection are similar, and establish that chronic inflammation by *H. pylori* causes superficial gastritis that may evolve to gastric atrophy and intestinal metaplasia in approximately half of patients, especially in patients suffering from severe inflammation [50, 51].

The weight of the dogs included in the study ranged between 6 and 32 kg, so the dose of *H. celastroides* extract administrated to the dogs ranged from 93.5 to 500 mg/kg in weight. The dose was determined according to the amount of plant used traditionally in humans (170 mg/kg), and taking as reference the doses of other plant extracts or plant preparation studies that were assayed for anti-*Helicobacter* activity such as: Amu-ru7 a Mongolian folk medicine composed from *Rhei rhizome, Hedychium spicatum, Radix auklandiae, Terminalia chebula,* Cape Jasmine fruit*, Piper longum* and Calcite (200 and 800 mg/kg) [52], *Calophyllum brasiliense* in Brazil (100 and 200 mg/kg) [53], and the Thai plant *Boesenbergia* (100 mg/kg) [54].

Results obtained from the experimental group indicated 33.3 % effectiveness, whereas effectiveness in the control group using the currently accepted standard treatment was 55 %. However, our current results showed no statistical difference in effectiveness between both groups. These figures do indicate how difficult is to eradicate this infection under the most controlled sanitized conditions, further indicating the importance of continued research for its eradication among both dogs and owners.

In comparison to effectiveness of the triple schema with amoxicillin-clarithromycin-omeprazole, we expected to obtain a similar proportion of effectiveness as that reported for humans in Mexican populations; however, our values were lower than that for assays among humans, which have shown a proportion of effectiveness of 65.5–89.7 % [33, 55, 56]. It may be that *H. celastroides* will demonstrate greater effectiveness. We propose a second pre-clinical assay with three groups of study, a first group with a higher dose of *H. celastroides* extract plus omeprazole, a second group with a lower dose plus omeprazole, and the third group with the standard triple schema plus *H. celastroides*. Taking into account the results obtained with *Nigella sativa*, which was administered to humans at doses of 1, 2 and 3 g in combination with omeprazole, showing frequencies of *H. pylori* eradication of 47.6, 66.7 and 47.8 % respectively, we expect the addition of omeprazole to improve effectiveness [57]. Likewise, in order to improve the eradication effect of the triple schema, *H. celastroides* will be added, anticipating similar results to those from the clinical assay, where the addition of cranberry juice improved the rate of *H. pylori* eradication from 80.0 % (omeprazole, amoxicillin and clarithromycin) to 95.2 % (omeprazole, amoxicillin, clarithromycin and cranberry juice) [58]. Some studies indicate that certain proton pump inhibitors (omeprazole), not only have an inhibitory effect on acid secretion, but also exert antibacterial activity in vitro, which is selective to *H. pylori* [59]. This antimicrobial activity is common to all benzimidazoles and absent in other anti-secretory drugs such as H2-antagonists [60, 61].

The safe therapeutic benefits of *H. celastroides* extract were demonstrated with the absence of renal and hepatic damage. There were 3 dogs with altered results in the *H. celastroides* group; however, levels of urea and creatinine had no clinical significance.

This is the first report indicating the prevalence of *Helicobacter spp.* infection in a Mexican canine population, and the first investigation to assay a medicinal plant in a canine population naturally infected with *Helicobacter spp.*

## Conclusions

*H. celastroides* is a non-toxic plant, so its use internally complies with GHS stipulations*.* The prevalence of dogs infected with *H. pylori* is very high, and zoonotic risk increases the need to treat this condition. The values indicating eradication of *Helicobacter spp* in the controlled preclinical trial of *H. celastroides* hydro-ethanolic extract and triple schema of amoxicillin-clarithromycin-omeprazole in naturally infected Mexican dogs showed no statistical difference. Both treatments were safe and well tolerated, when taken orally.

## Additional file

Additional file 1: HPTLC profiling of H. celastroides and H. excelsa for alkaloids and triterpenes analysis. (DOCX 239 kb)

## Abbreviations

ALT: Alanine aminotransferase
ANOVA: Analysis of variance
APAC: Asociación Protectora de Animales Philip Kahan
AST: Aspartate transaminase
CCl_4_: Carbon tetrachloride
CEIB: Centro de Investigación en Biotecnología
CIB: Centro de Investigaciones Biológicas
COFEPRIS: Comisión Federal para la Protección Contra los Riesgos Sanitarios
EtOH: Ethyl alcohol
GHS: Globally harmonized system
H2: Histamine
HFS-30: Normal fibroblast cell line
HPTLC: High performance thin layer chromatography
INSP: Instituto Nacional de Salud Pública
KB: Nasopharyngeal cancer cell line
LD_50_: Median lethal dose
MALT: Mucosa-associated lymphoid tissue
MCF-7: Breast cancer cell line
MeOH: Methanol
MIC: Minimum inhibitory concentration
NIH: National Institute of Health
NOAEL: Non-observed-adverse-level
SEM: Standard error of the mean
UAEM: Universidad Autónoma del Estado de Morelos
US: United States

## References

[1] Castillo-Campos G, Medina AM (2005). Flora de Veracruz Hippocrateaceae.

[2] Reyes-Chilpa R, Jimenez-Estrada M, Cristobal-Telésforo E, Torres-Colín L, Villavicencio MA, Pérez-Escandón BE, Mercado-González R (2003). Natural insecticides from *Hippocratea excelsa* and *Hippocratea celastroides*. Econ Bot.

[3] Monroy-Ortíz C, Castillo-España P (2000). Plantas Medicinales Utilizadas en el Estado de Morelos.

[4] Sanabria-Diago OL (1986). El Uso y Manejo Forestal en la Comunidad Xul en el Sur de Yucatán. Etnoflora Yucatense.

[5] Argueta-Villamar A, Cano-Asseleih LM, Rodarte ME (1994). Atlas de las Plantas de la Medicina Tradicional Mexicana.

[6] Soto Núñez JC, Sousa M (1995). Plantas Medicinales de la Cuenca del Río Balsas.

[7] Castillo EP, Monroy OC: Plantas Medicinales Utilizadas en el Estado de Morelos. Comisión Nacional para el Conocimiento y Uso de la Biodiversidad. Universidad Autónoma del Estado de Morelos: México Press, 2007

[8] González AG, Bazzochi IL, Ravelo G, Luis GJ (1989). Triterpenos de *Hippocratea celastroides* (Celastraceae). Rev Latinoam Quím.

[9] Jiménez-Estrada MR, Reyes-Chilpa S, Hernández-Ortega E, Cristobal-Telésforo E, Torres Colín L, Jankowsky CK, Aumelas A, Van Calesteren MR (2000). Two novel dielsalder adducts from *Hippocratea celastroides* roots and their insecticidal activity. Can J Chem.

[10] Hinojosa II, Quiróz MA, Álvarez IR, Castañeda PE, Villarreal ML, Taketa AC (2014). Anti-*Helicobacter pylori*, gastroprotective, anti-inflammatory and cytotoxic activities of methanolic extracts of five different populations of *Hippocratea celastroides* collected in Mexico. J Ethnopharmacol.

[11] Tan VP, Wong BC (2011). *Helicobacter pylori* and gastritis: Untangling a complex relationship 27 years on. J Gastroenterol Hepatol.

[12] Kuster JG, Van Vliet AH, Kuipers EJ (2006). Pathogenesis of *Helicobacter pylori* infection. Clin Microbiol Rev.

[13] Van den Bulck K, Decostere A, Baele M, Vandamme P, Mast J, Ducatelle R, Haesebrouck F (2006). *Helicobacter cynogastricus* sp. Nov., isolated from the canine gastric mucosa. Int J Syst Evol Microbiol.

[14] Buczolits S, Hirt R, Rosengarten R, Busse HJ (2003). PCR-based genetic evidence for occurrence of *Helicobacter pylori* and novel *Helicobacter* species in the canine gastric mucosa. Vet Microbiol.

[15] Jalava K, De Ungria MC, O’Rourke J, Lee A, Hirvi U, Hänninen ML (1999). Characterization of *Helicobacter felis* by pulsed-field gel electrophoresis, plasmid profiling and ribotyping. Helicobacter.

[16] Jalava K, Kaartinen M, Ultriainen M, Happonen I, Hänninen ML (1997). *Helicobacter salomonis* sp. Nov., a canine gastric *Helicobacter sp.* related to *Helicobacter felis* and *Helicobacter bizzozeronii*. Int J Syst Bacteriol.

[17] Hermanns W, Kregel K, Breuer W, Lechner J (1995). *Helicobacter* like organisms: histopathological examination of gastric biopsies from dogs and cats. J Comp Pathol.

[18] Happonen I, Linden J, Saari S, Karjalainem M, Hänninen ML, Jalava K, Westermarck E (1998). Detection and effects of helicobacers in healthy dogs and dogs with signs of gastritis. J Am Vet Med Assoc.

[19] Neiger R, Simpson KW (2000). Helicobacter infection in dogs and cats: facts and fiction. J Vet Intern Med.

[20] Hwang CY, Han HR, Youn HY (2002). Prevalence and clinical characterization of gastric *Helicobacter* species infection of dogs and cats in Korea. J Vet Sci.

[21] Sapierzynski R, Malicka E, Bielecki W, Sendecka H (2003). The presence of Helicobacter-like microorganisms in the gastric mucosa in dogs. Pol J Vet Sci.

[22] Haesebrouck F, Pasmans F, Flahou B, Chiers K, Baele M, Meyns T, Decostere A, Ducatelle R (2009). Gastric helicobacters in domestic animals and non human primates and their significance for human health. Clin Microbiol Rev.

[23] Castiglioni V, VailatiFacchini R, Mattiello S, Luini M, Gualdi V, Scanziani E, Recordati C (2012). Enterohepatic *Helicobacter spp.* in colonic biopsies of dogs: molecular, histopathological and immunohistochemical investigations. Vet Microbiol.

[24] Lux CN, Archer TM, Lunsford KV (2012). Gastroesophageal reflux and laryngeal dysfunction in a dog. J Am Vet Med Assoc.

[25] Kivistö R, Linros J, Rossi M, Rautelin H, Hänninen ML (2010). Characterization of multiple *Helicobacter bizzozeronii* isolates from a finish patient with severe dyspeptic symptoms and chronic active gastritis. Helicobacter.

[26] Hänninen ML, Happonen I, Jalava K (1998). Tansmission of canine gastric *Helicobacter salomonis* infection from dam to offspring and between puppies. Vet Microbiol.

[27] Svec A, Kordas P, Pavlis Z, Novotny J (2000). High prevalence of *Helicobacter heilmannii*- associated gastritis in a small, predominantly rural area; further evidence in support of a zoonosis?. Scand J Gastroenterol.

[28] Recordati C, Gualdi V, Tosi S, Facchini RV, Pengo G, Luini M, Simpson KW (2007). Detection of *Helicobacter spp* DNA in the oral cavity of dogs. Vet Microbiol.

[29] Azevedo NF, Almeida C, Fernández I, Cerqueira L, Dias S, Keevil CW, Vieira MJ (2008). Survival of gastric and enterohepatic *Helicobacter spp.* in water: implications for transmission. Appl Environ Microbiol.

[30] Anacleto TP, Lopes LR, Andreollo NA, BernisFilho WO, Resck MC, Macedo A (2011). Studies of distribution and recurrence of *Helicobacter spp*. Gastric mucosa of dogs after triple therapy. Acta Cir Bras.

[31] GPC (Guía de Practica Clínica). SS-ISO-08 RR-Guía de Referencia Rápida. Consejo de Salubridad General. Secretaría de Salud, 2011.

[32] Greenberg ER, Anderson GL, Morgan DR, Torres J, Chey WD, Bravo LE, Dominguéz RL, Ferreccio C, Herrero R, Lazcano-Ponce EC, Meza-Montenegro MM, Peña R, Peña EM, Salazar-Martínez E, Correa P, Martínez ME, Valdivieso M, Goodman GE, Crowley JJ, Baker LH (2011). 14-day triple, 5-day concomitant, and 10-day sequential therapies for *Helicobacter pylori* infection in seven Latin American sites: a randomized trial. Lancet.

[33] Garza-González E, Giasi González E, Martínez Vázquez MA, Pérez Pérez GI, González GM, Maldonado Garza HJ, Bosques Padilla FJ (2007). *Helicobacter pylori* eradication and its relation to antibiotic resistance and CYP2C19 status. Rev Esp Enferm Dig.

[34] Kato S, Ozawa K, Sekine H, Ohyauchi M, Shimosegawa T, Minoura T, Linuma K (2005). *Helicobacter heilmannii* infection in a child after successful eradication of *Helicobacter pylori*: case report and review of literature. J Gastroenterol.

[35] Simpson KW, Strauss-Ayali D, McDonough PL, Chang YF, Valentine BA (1999). Gastric function in dogs with naturally acquired gastric *Helicobacter spp*. infection. J Vet Intern Med.

[36] Cornetta AM, Simpson KW, Strauss-Ayali D, McDonough PL, Gleed RD (1998). Use of a [13C] urea breath test for detection of gastric infection with *Helicobacter spp.* in dogs. Am J Vet Res.

[37] Assob JC, Kamga HL, Nsafha DS, Njunda AL, Nde PF, Asongalem EA, Njouendou AJ, Sandjon B, Penlap VB (2011). Antimicrobial and toxicological activities of five medicinal plant species from Cameroon traditional medicine. BMC Complement Altern Med.

[38] Li LJ, Yang YG, Zhang ZL, Nie SF, Li Z, Li F, Hua HY, Hu YJ, Zhang HS, Guo YB (2007). Protective effects of medical ozone combined with traditional Chinese medicine against chemically-induced hepatic injury in dogs. World J Gastroenterol.

[39] Yi H, Thurberg BL, Curtis S, Austin S, Fyfe J, Koeberl DD, Kishnani PS, Sun B (2012). Characterization of a canine model of glycogen storage disease type IIIa. Dis Model Mech.

[40] Pei Z, Zhang X (2014). Methamphetamine intoxication in a dog: case report. BMC Vet Res.

[41] Borges M, Marini Filho R, Laposy CB, Guimarães-Okamoto PT, Chaves MP, Vieira AN, Melchert A (2013). Nonsteroidal anti-inflammatory therapy: changes on renal function of healthy dogs. Acta Cir Bras.

[42] Copplestone JF (1988). The development of the WHO recommended classification of pesticides by hazard. Bull World Health Organ.

[43] WHO (1987). Principles for the Safety Assessment of Food Additives and Contaminants in Food.

[44] Hermenean A, Popescu C, Ardelean A, Stan M, Hadaruga N, Mihali CV, Costache M, Dinischiotu A (2012). Hepatoprotective effects of *Berberis vulgaris* L. extract/βcyclodextrin on carbon tetrachloride-induced acute toxicity in mice. Int J Mol Sci.

[45] Ho WY, Yeap SK, Ho CL, Abdul Rahim R, Alitheen (2012). Hepatoprotective activity of *Elephantopus scaber* on alcohol-induced liver damage in mice. Evid Based Complement Alternat Med.

[46] Cheesbrough M: Medical Laboratory Manual for Tropical Countries, Microbiology EIBS. Low Price Edition; 1985

[47] Eaton KA, Dewhirst FE, Paster BJ, Tzellas N, Coleman BE, Paola J, Sherding R (1996). Prevalence and varieties of Helicobacter species in dogs from random sources and petdogs: animal and public health implications. J Clin Microbiol.

[48] Polanco R, Salazar V, Reyes N, García-Amado MA, Michelangeli F, Contreras M (2011). High prevalence of DNA from non-*H. pylori* helicobacters in the gastric mucosa of Venezuelan pet dogs and its histological alterations. Rev Inst Med Trop Sao Paulo.

[49] Happonen I, Saari S, Castren L, Tyni O, Hanninen ML, Westermarck E (1996). Comparison of diagnostic methods for detecting gastic Helicobacter-like organisms in dogs and cats. J Comp Pathol.

[50] Dîrnu R, Sexureanu FA, Neamtu C, Totolici BD, Pop OY, Mitrut P, Malaescu DG, Mogoanta L (2012). Chronic gastritis with intestinal metaplasia: clinical-statistical, histological and immunohistochemical study. Rom J Morphol Embryol.

[51] Kiopers EJ, Uyterlinde AM, Peña AS, Hazenberg HJ, Bloemena E, Lindeman J, Klinkenberg-Knol EC, Meuwissen SG (1995). Increase of *Helicobacter pylori* associated corpus gastritis during acid suppressive therapy: implications for long-term safety. Am J Gastroenterol.

[52] Bai CL, Osaki T, Yonezawa H, Hanawa T, Zamac CM Kurata S, Kamiya S, Tanaka H (2010). *In vitro* and *In vivo* effects of the Mongolian drug Amu-ru 7 on *Helicobacter pylori* growth and viability. Microbiol Immunol.

[53] Souza C, Beserra A, Martins DC, Real VV, Santos RA, Rao VS, Silva RM, Martins DT (2009). *In vitro* and *in vivo* anti-*Helicobacter pylori* activity of *Calophyllum brasiliense Camb*. J Ethnopharmacol.

[54] Mahady GB, Bhamarapravati S, Adeniyi BA, Doyle B, Locklear T, Slover C, Pendland SL (2006). Traditional Thai medicines inhibit *Helicobacter pylori in vitro* and *in vivo:* Support for ethnomedical use. Ethnobot Res Appl.

[55] González-Huezo MS, Rojas-Sámchez A, Rosales-Solís AA, Miranda-Cordero RM, Hinojosa-Ruiz A, Mejía-García E, Cruz-González EG (2012). *Helicobacter pylori* eradication frequency with the conventional triple therapy in adult patients at the Centro Médico Issemym. Rev Gastroenterol Mex.

[56] Dehesa M, Larisch J, Dibildox M, Di Silvio M, López LH, Ramirez-Barba E, Torres J (2002). Comparison of three 7-day pantoprazole-based *Helicobacter pylori* eradication regimens in a Mexican population with high metronidazole resistance. Clin Drug Investig.

[57] Salem EM, Yar T, Bamosa AO, Al-Quorain A, Yasawy MI, Alsulaiman RM, Randhawa MA (2010). Comparative study of *Nigella sativa* and triple therapy in eradication of *Helicobacter pylori* in patients with non-ulcer dyspepsia. Saudi J Gastroenterol.

[58] Shmuely H, Yahav J, Samra Z, Chodixk G, Koren R, Niv Y, Ofek I (2007). Effect of cranberry juice on eradication of *Helicobacter pylori* in patients treated with antibiotics and a proton pump inhibitor. Mol Nutr Food Res.

[59] Figura N, Crabtree JE, Dattilo M (1997). *In vitro* activity of lansoprazole against *Helicobacter pylori*. J Antimicrob Chemother.

[60] Dattilo M, Figura N (1998). *Helicobacter pylori* infection, chronic gastritis, and proton pump inhibitors. J Clin Gastroenterol.

[61] Nakao M, Tada M, Tsuchimori K, Uekata M (1995). Antibacterial properties of lansoprazole alone and in combination with antimicrobial agents against *Helicobacter pylori*. Eur J Clin Microbiol Infect Dis.

[62] OECD (2008). OECD Guidelines for the Testing of Chemicals. Repeated Dose 28-day Oral Toxicity Study in Rodent. The Guideline No. 407, OECD Guidelines for the testing of Chemicals.

[63] OECD (2001). OECD Guideline for Testing of Chemicals. Acute Oral Toxicity – Fixed Dose Procedure. Test Guideline No. 420, OECD Guidelines for the Testing of Chemicals.

