# Supplementary material for: Preclinical evaluation of anti-Helicobacter spp. activity of Hippocratea celastroides Kunth and its acute and sub-acute toxicity
Source: BMC Complement Altern Med. 2016 Nov 8;16:445. doi: 10.1186/s12906-016-1412-6 (PMC5101718; doi:10.1186/s12906-016-1412-6)
Supplement: Additional file 1: — HPTLC profiling of H. celastroides and H. excelsa for alkaloids and triterpenes analysis. (DOCX 239 kb) [file 12906_2016_1412_MOESM1_ESM.docx]

HPTLC profiling of *H. celastroides* and *H. excelsa* for alkaloids and triterpenes analysis


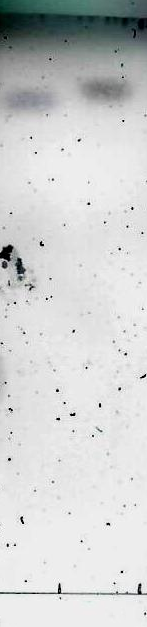

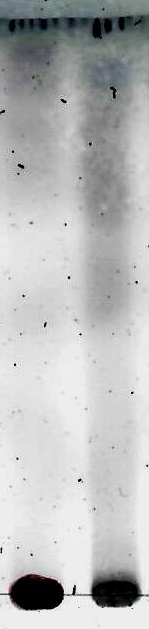

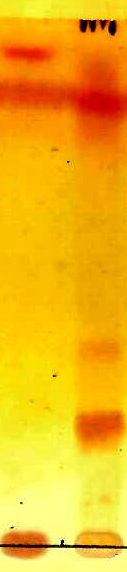


B)

A)

6

5

4

3

2

1

1- *H. excelsa* MeOH extract (Wagner reagent)

2- *H. celastroides* hydroalchoholic extract (Wagner reagent)

3- *H. excelsa* MeOH extract (Vanillin/H_2_SO_4_/heat)

4- *H. celastroides* hydroalchoholic extract (Vanillin/H_2_SO_4_/heat)

5- Ursolic acid (Vanillin/H_2_SO_4_/heat)

6- Diosgenin (Vanillin/H_2_SO_4_/heat)

**CHROMATOGRAFIC CONDITIONS:**

A) HPTLC Si 60

Mobile phase CHCl_3_:MeOH:HCOOH (100:40:10)

B) HPTLC Si 60

Mobile phase CHCl_3_:MeOH (8:2)
